# Leukocyte Depletion During CPB: Effects on Inflammation and Lung Function

**DOI:** 10.1007/s10753-013-9730-z

**Published:** 2013-10-04

**Authors:** Célio Gomes de Amorim, Luiz Marcelo Sá Malbouisson, Francisco Costa da Silva, Alfredo Inácio Fiorelli, Caroline Kameio Fernandes Murakami, Maria José Carvalho Carmona

**Affiliations:** 1 Discipline of Anesthesiology—Instituto do Coração, Faculdade de Medicina da Universidade de São Paulo, São Paulo, SP Brazil; 2 Dom Almir Marques Ferreira, 644, Morada da Colina, Uberlândia, Minas Gerais Brazil

**Keywords:** coronary artery bypass surgery, cardiopulmonary bypass, leukocyte reduction filtrations, pulmonary function, systemic inflammatory response

## Abstract

Cardiopulmonary bypass (CPB) is related to inflammatory response and pulmonary dysfunction. The aim of this study was to evaluate the effects of CPB leukocyte filtration on inflammation and lung function after coronary artery bypass grafting (CABG). A prospective randomized study was performed to compare CABG patients undergoing CPB leukocyte filtration (*n* = 9) or standard CPB (*n* = 11). Computed tomography, oxygenation, leukocyte count, hemodynamic data, PaO_2_/FiO_2_, shunt fraction, interleukins, elastase, and myeloperoxidase were evaluated. Data were analyzed using two-factor ANOVA for repeated measurements. The filtered group showed lower neutrophil counts up to 50 min of CPB, lower shunt fraction up to 6 h after surgery, and lower levels of IL-10 at the end of surgery (*p* < 0.05). There was no statistically significant difference between groups related to other parameters. Leukodepletion during CPB results in neutrophil sequestration by a short time, decreased IL-10 serum levels, and lower worsening of lung function only temporarily.

## INTRODUCTION

Changes in pulmonary function observed after coronary surgery with cardiopulmonary bypass (CPB) are related to increases in postoperative morbidity and mortality, principally if prolonged mechanical ventilation is required [1]. The systemic and pulmonary inflammatory response plays an important role in this multifactorial process [2–4]. Leukocyte activation and consequent alterations in endothelial integrity lead to changes in vascular permeability and resistance, which can worsen the ventilation/perfusion ratio and interfere with postoperative outcome [2, 3, 5]. Different kinds of pharmacological therapy, CPB circuits, and lung-protective mechanical ventilation strategies have been used in order to decrease the inflammatory response, although the mechanism of each is incompletely understood [2, 4, 6]. Additionally, it is necessary to consider the secondary effects on pulmonary and systemic microcirculation.

Leukocyte depletion during CPB, although controversial [7], can reduce unwanted inflammatory response [8, 9], decreasing IL-6 and IL-8 activity, with possible improvement in hemodynamic parameters and the oxygenation index [9–11]. On the other hand, the effect of filtration on anti-inflammatory levels of interleukins such as IL-10 and IL-1rA is not very clear. As IL-10 anti-inflammatory activity is partially triggered by downregulation of leukocyte expression of the major histocompatibility class II molecule (HLA-DR) [12], the level of this mediator can reflect the balance of pro- and anti-inflammatory cytokine expression, an aspect which deserves better understanding [13]. In inflammatory conditions, increased IL-10 levels can lead to a worsened clinical outcome [14, 15], although individual characteristics of IL-10 secretion, as a consequence of genetic polymorphism in its promoter, have been implicated in variable patterns of its expression [14]. We, therefore, hypothesized that leukocyte filtration during CPB could decrease the magnitude of systemic inflammatory response in individuals undergoing coronary artery bypass grafting, contributing to better postoperative morbidity.

The aims of this study were to evaluate if the leukocyte filter could reduce the inflammatory response proportionally to reduction in WBC count and what could be the impact on postoperative lung function, accessed by mediators of pro- and anti-inflammatory activity, extravascular lung water, loss of aeration, and oxygenation.

## METHODS

After the approval statement of the ethics committee for this study (Ethical Committee No. 216/04), provided by the ethics committee for review of research projects (CAPPesq) of Clinics Hospital of the Medical School of the University of São Paulo, São Paulo, Brazil, a prospective randomized study was performed for evaluation of individuals undergoing CABG who had their physical state classified as PII or PIII, according to the American Society of Anesthesiologists (ASA) [16]. Surgical risk was stratified according to the Parsonnet criteria [17], and only those individuals considered to be of low to moderate risk were admitted. Subjects older than 70 years or those presenting with body mass index (BMI) over 35 kg/m^2^, with congestive heart failure (CHF) greater than class III (NYHA) [18], or with left ventricle ejection fraction less than 40 %; who had recently submitted to other surgery; who had creatinine levels ≥1.4 mg/dL; or who were using oral anticoagulants were all excluded.

### Anesthesia Procedures

After signing the free and clarified consent form, subjects were evaluated with respect to demographic data, personal history, and blood samples. Patients received midazolam at a dose of 0.1 to 0.2 mg/kg orally, 30 min before surgery, to a maximum dose of 15 mg. After admission to the operating room, they were monitored through pulse oximetry, five-lead electrocardiogram, and continuous ST segment analysis (Siemens, Berlin, Germany). Peripheral venipuncture was obtained in the upper limb with a 14G or 16G catheter. Invasive blood pressure monitoring was performed after radial artery puncture with a 20G catheter, using a pressure transducer, and verifying attainment of the pressure curve (Siemens monitor SC 9000 Infinity XL, Munich, Germany). After preoxygenation with 100 % oxygen, intravenous infusion with 0.5 μg/kg sufentanil and 0.2 mg/kg etomidate was performed, and muscle relaxation was obtained with 0.1–0.2 mg/kg pancuronium bromide, followed by manual mask ventilation and tracheal intubation with a canula of suitable diameter. Mechanical ventilation was performed in a circular valve system with a carbon dioxide absorber, according to NBR/ABNT No. 10012 (Brazilian Association of Technical Standards), by a microprocessed electronic fan from a set of anesthesia equipment (Cicero®, Dräger and Siemens Company, Lübeck, Germany). It was adjusted to a volume chain (VC) of 8 mL/kg, FiO_2_ of 0.6, I/E ratio of 1:2, respiratory rate (RR) of 12 breaths/min (bpm), and positive end-expiratory pressure (PEEP) of 5 cm H_2_O and adjusted to maintain PETCO_2_ between 35 and 40 mmHg. Anesthesia was maintained with isoflurane inhalation adjusted between 0.5 and 1.0 MAC, and continuous infusion of sufentanil at 0.5 μg/kg h. During CPB, ventilation was interrupted and hypnosis was maintained by propofol infusion at a target-controlled concentration of 2.5 μg/mL.

After general anesthesia induction, nasopharynx temperature sensor placement, bladder catheterization for diuresis control, and pulmonary artery catheterization (7.5F catheter with thermal filament—CCO catheter, Baxter Edwards Critical Care, Irvine, CA, USA) connected to a Vigilance monitor (Baxter Edwards Critical Care, Irvine, CA, USA) were carried out.

### Randomization and CPB Management

For the CPB pump, a non-heparinized roller circuit (Braille, São Jose do Rio Preto, Brazil) was used, filled with Ringer's solution to a total volume of 1,500 mL, and maintained near an average flux of 3,500 to 4,500 (3.5–4.5 L/m^2^ min), according to immediate need. Mild hypothermia between 28 and 32 °C was maintained; a membrane-type oxygenator was also used. Heparin (500 U/kg) was applied as an anticoagulant for CPB installation, and protamine was used to reverse the heparin's effect. The antifibrinolytic agent used was epsilon-aminocaproic acid (80 mg/kg); cardioplegia consisted of standard cardioplegic solution, added to the mixture of blood drained from the patient with priming contained in the reservoir.

Individuals were allocated randomly into the “control” or “filtered” groups according to simple randomization occurring immediately before surgery. In the filtered group, an additional leukocyte filter (Pall Biomedical Product, East Hills, NY, USA) was placed alongside the standard filter, which was clamped during CPB. In the control group, only the standard filter was used.

At the time of rewarming under CPB, inotropes or vasodilators were introduced by the anesthesiologist. At the end of the surgery, patients were transferred to the surgical intensive care unit.

### Data Collection

A fast spiral thoracic CT scan (Aquilion 64, Toshiba Medical Systems, Otawara, Japan) was obtained during a 10-s period of apnea at the end of a normal expiration, with 10-mm cuts without interval, preoperatively and on the first postoperative day, to measure lung density along the sectional area, with evaluation of well, poorly, or non-aerated regions of the lung.

Hemodynamic data and the PaO_2_/FiO_2_ index value were obtained preoperatively (Pre-op.), after induction of anesthesia (After induction), 5 min before CPB (Beginning of CPB), 5 min after End of CPB (End of CPB), during skin suturing (End of surgery), and at 6 (6 h P.O.), 12 (12 h P.O.), and 24 h (24 h P.O.) after surgery. Intrapulmonary shunt fraction (Qs/Qt), TNF-α, IL-1β, IL-6, IL-8, IL-10, IL-1rA, elastase, and myeloperoxidase (MPO) were also evaluated at the following times: After induction, Beginning of CPB, End of CPB, End of surgery, and at 6, 12, and 24 h P.O. Cell counts were performed at the following times: after induction, after 5 min of CPB (5 min CPB), after 25 min of CPB (25 min CPB), after 50 min of CPB (50 min CPB), during suturing (End surgery), and at 12 and 24 h P.O. The presence and type of any infection were recorded postoperatively.

### Sample Size Calculation and Statistical Analysis

The sample size was calculated based on the assumption that, when CPB started, the first white blood cell count would have a numerical reduction of at least 30 % pre-CPB, which, in turn, was considered taking as reference an average of 5,000 cells, with a standard deviation of 900 [19, 20]. During CPB, still in relation to the first cell count, a confidence interval of 95 % was considered, with an interval length of 600, which generated an *n* of 8 individuals in the filtered group.

Quantitative and demographic variables were descriptively presented in tables with means and standard deviations. Where indicated, *t* test was carried out for independent samples.

Analysis of variance (ANOVA) for repeated measurements was used to verify differences between groups. The method of ANOVA included the factor group, time, and interaction between time and group. For those tests which showed statistically significant differences (*p* < 0.05), we followed up with the Student–Newman–Keuls test for multiple comparisons between groups and times.

## RESULTS

**Fig. 1:**
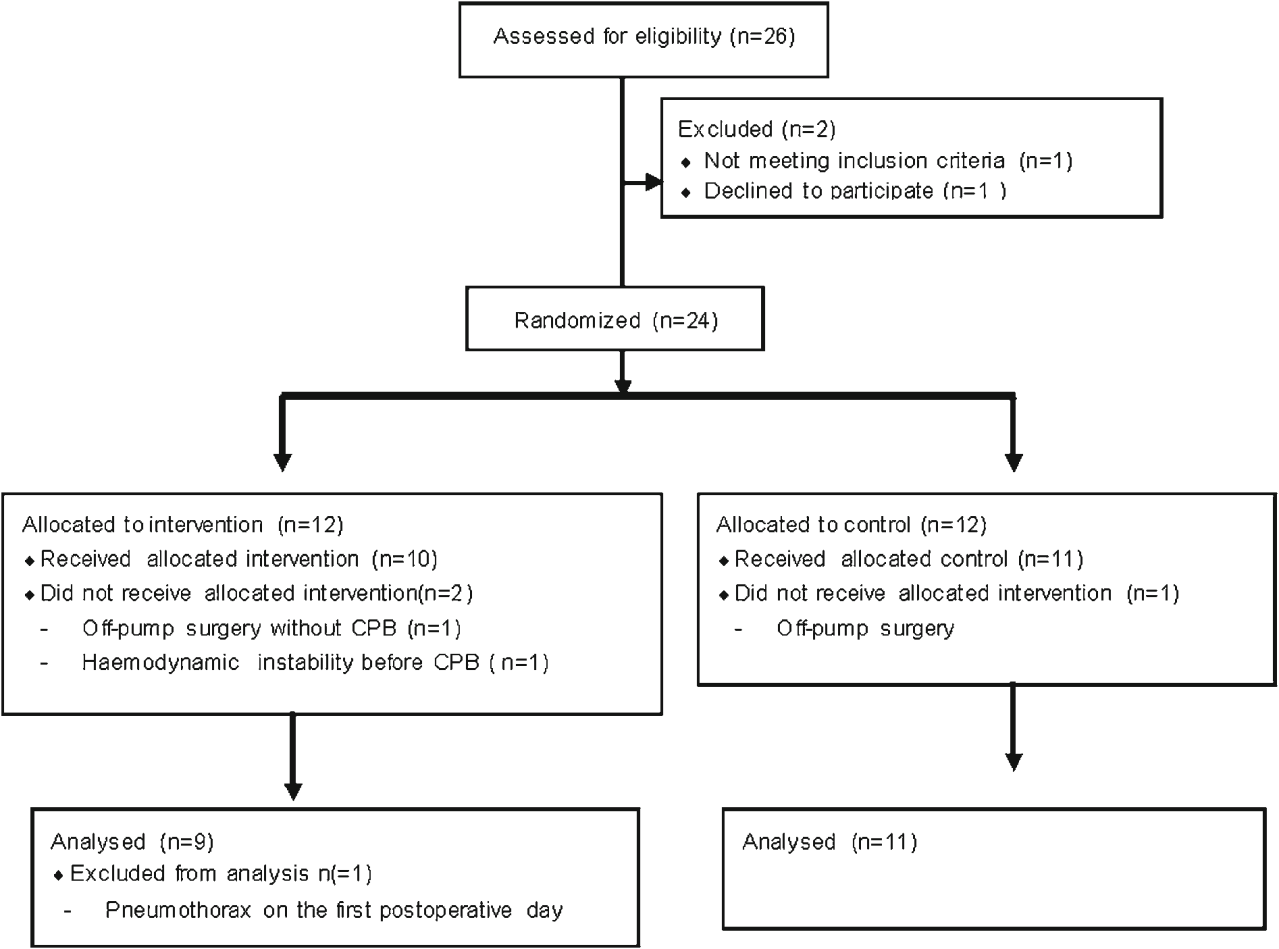
Flow diagram.

**Fig. 2:**
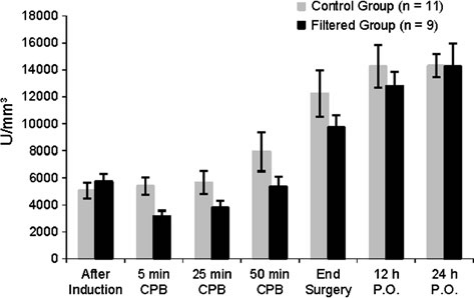
Changes in leukocyte counts (mean ± SEM). Comparison between groups by two-factor ANOVA for repeated measures (*p* > 0.05).

**Fig. 3:**
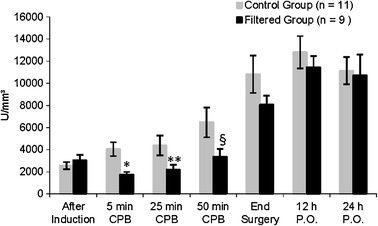
Changes in differential neutrophil counts (mean ± SEM). Comparison between groups by two-factor ANOVA for repeated measures. Statistically significant difference between groups: *p = 0.002; **p = 0.032; §*p* = 0.046.

A total of 26 individuals were initially assessed for eligibility of participation, but only 20 completed the study: 11 in the control group and 9 in the filtered group (Fig. 1). One did not meet the inclusion criteria during the interview. Another refused to participate after the interview. Twenty-four individuals were randomized: 12 to the control group and 12 to the filtered group. Two individuals in the filtered group did not receive the allocated intervention. Another was excluded from the final analysis, which resulted at the end in nine individuals. In the control group, 1 individual was excluded from the final analysis, resulting in 11 individuals. The groups were comparable concerning demographic data, length of CPB, and hemodynamic data (Tables 1 and 2). Although the total leukocyte count did not present explicit differences between groups during CPB (*p* = 0.084), we observed a greater decrease in the neutrophil count in the filtered group than in the control group, from the beginning to 50 min of CPB (*p* = 0.036), as shown in Figs. 2 and 3.

**Table 1:** Demographic Data and Procedure Times (Mean ± SD)

Variable	Control group	Filtered group	*p* value
Gender (M/F)	7:4	5:4	
Age (years)	61.00 ± 10.58	65.00 ± 9.26	0.336^a^
BMI (kg/cm^2^)	27.65 ± 3.20	27.60 ± 4.48	0.976^a^
Weight (kg)	77.76 ± 1.54	70.17 ± 14.82	0.243^a^
Height (m)	1.66 ± 0.07	1.60 ± 0.08	0.106^a^
Length of CPB (min)	104.64 ± 27.76	86.78 ± 19.58	0.161^a^
Length of surgery (min)	392.50 ± 70.30	379.50 ± 91.00	0.520^a^
Time to extubation (min)	463.18 ± 188.98	421.67 ± 122.98	0.578^a^

^a^ *t* test for independent samples

**Table 2:** Hemodynamic Data for the Control and Filtered Groups (Mean ± SD)

	After induction	Beginning of CPB	End of CPB	End of surgery	6 h P.O.	12 h P.O.	24 h P.O.	*p* value
HR (bpm)								0.697^a^
Control	69 ± 14	79 ± 15	97 ± 13	97 ± 13	100 ± 14	93 ± 10	100 ± 12	0.001^b^
Filtered	67 ± 14	74 ± 14	97 ± 11	101 ± 12	95 ± 15	94 ± 11	98 ± 18	
MAP (mmHg)								0.626^a^
Control	69.45 ± 15.57	63.55 ± 8.44	63.36 ± 8.33	746.09 ± 4.88	81.64 ± 8.94	85.27 ± 6.80	74.55 ± 7.22	0.001^b^
Filtered	72.78 ± 14.95	70.22 ± 12.93	65.67 ± 7.14	63.33 ± 6.60	84.67 ± 11.07	80.44 ± 11.06	80.92 ± 7.57	
MPAP (mmHg)								0.772^a^
Control	21.82 ± 7.70	20.70 ± 6.63	21.55 ± 4.76	23.91 ± 9.47	24.09 ± 7.89	23.09 ± 7.61	19.00 ± 4.49	0.432^b^
Filtered	19.89 ± 6.58	18.89 ± 4.83	24.33 ± 7.83	23.89 ± 6.13	21.78 ± 8.07	20.22 ± 8.38	20.78 ± 7.43	
PCWP (mmHg)								0.964^a^
Control	16.45 ± 11.44	13.40 ± 5.95	16.64 ± 8.76	12.00 ± 3.50	13.22 ± 3.77	11.55 ± 5.72	11.30 ± 3.53	0.265^b^
Filtered	17.15 ± 8.13	13.77 ± 5.69	16.08 ± 5.85	17.07 ± 6.81	13.85 ± 6.18	12.77 ± 6.77	13.00 ± 5.82	
CO (L/min)								0.196^a^
Control	4.67 ± 1.59	5.29 ± 1.37	8.10 ± 2.72	7.36 ± 1.85	6.55 ± 1.26	6.12 ± 1.31	7.13 ± 1.19	0.001^b^
Filtered	3.97 ± 1.13	5.08 ± 1.68	7.04 ± 1.71	5.22 ± 0.76	5.07 ± 1.15	5.20 ± 1.27	5.87 ± 0.92	
CI (L/min/m^2^)								0.228^a^
Control	2.50 ± 0.77	2.94 ± 0.70	4.52 ± 1.48	4.29 ± 1.08	3.52 ± 0.58	3.43 ± 0.56	3.72 ± 0.46	0.001^b^
Filtered	2.29 ± 0.55	2.83 ± 0.89	4.18 ± 1.23	3.06 ± 0.52	2.97 ± 0.66	3.06 ± 0.70	3.41 ± 0.50	
SVR (dyn s/cm^5^)								0.777^a^
Control	1,343 ± 666	833 ± 230	675 ± 278	700 ± 260	945 ± 219	1,012 ± 201	872 ± 182	0.001^b^
Filtered	1,388 ± 488	1,195 ± 587	590 ± 114	822 ± 163	1,171 ± 283	1,149 ± 271	951 ± 159	
PVR (dyn s/cm^5^)								0.596^a^
Control	126 ± 74	112 ± 48	107 ± 53	102 ± 29	90 ± 33	142 ± 88	91 ± 24	0.242^b^
Filtered	105 ± 56	99 ± 31	96 ± 47	121 ± 36	140 ± 87	118 ± 52	99 ± 37	
SVRI (dyn s/cm^5^)								0.747^a^
Control	2,234 ± 705	1,502 ± 327	1,132 ± 389	1,299 ± 481	1,807 ± 490	1,820 ± 341	1,640 ± 417	0.001^b^
Filtered	2,267 ± 725	1,932 ± 1,044	1,013 ± 195	1,412 ± 280	1,997 ± 419	1,946 ± 446	1,613 ± 280	
PVRI (dyn s/cm^5^)								0.550^a^
Control	227 ± 117	203 ± 85	181 ± 95	188 ± 58	170 ± 69	244 ± 142	165 ± 54	0.422^b^
Filtered	173 ± 85	167 ± 44	169 ± 92	210 ± 65	239 ± 143	184 ± 81	171 ± 63	

*p* values = comparison between groups by two-factor ANOVA for repeated measures

*HR* heart rate, *MAP* mean arterial pressure, *MPAP* mean pulmonary arterial pressure, *PCWP* pulmonary capillary wedge pressure, *CO* cardiac output, *CI* cardiac index, *SVR* systemic vascular resistance, *PVR* pulmonary vascular resistance, *SVRI* systemic vascular resistance index, *PVRI* pulmonary vascular resistance index

^a^Difference between groups

^b^Variation over time by the Student–Newman–Keuls test

**Fig. 4:**
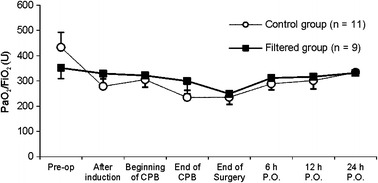
PaO_2_/FiO_2_ ratios in the different groups (mean ± SEM). Comparison between groups by two-factor ANOVA for repeated measures: *p < 0.05 (statistically significant difference between groups).

**Fig. 5:**
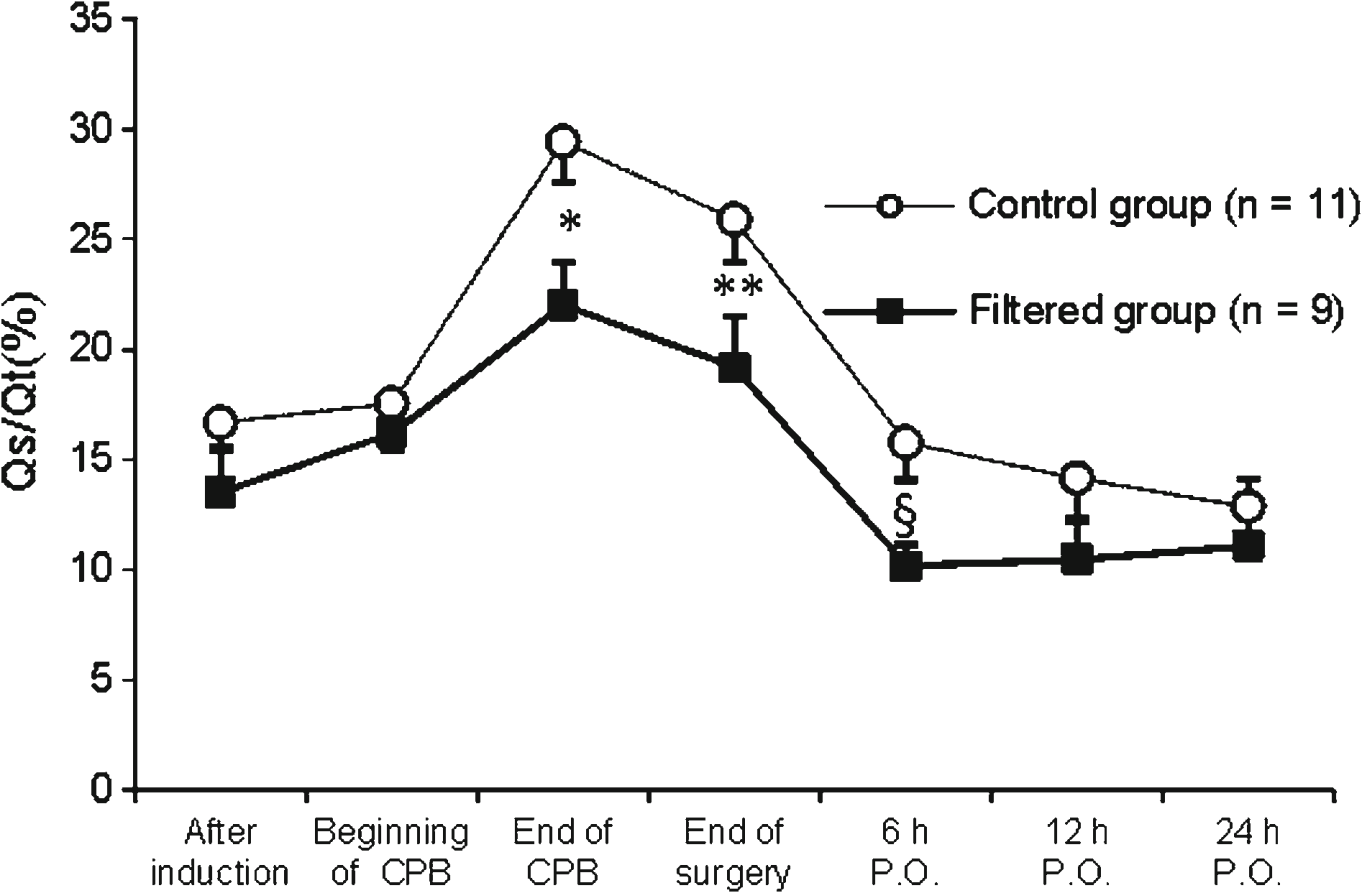
Pulmonary shunt fractions in the different groups (mean ± SEM). Comparison between groups by two-factor ANOVA for repeated measures. Statistically significant difference between groups: *p = 0.005; **p = 0.006; §*p* = 0.045.

Regarding respiratory data, both groups presented slight reductions in PaO_2_/FiO_2_, from the induction of anesthesia to the end of surgery, with a gradual restoration of normal values up to 24 h after the end of surgery (Fig. 4), without differences between the groups. However, from the end of CPB to 6 h after surgery, the rise in Qs/Qt was less in the filtered group than in the control group (*p* = 0.040), with a tendency to return to baseline in both groups on the first postoperative day (Fig. 5).

**Fig. 6:**
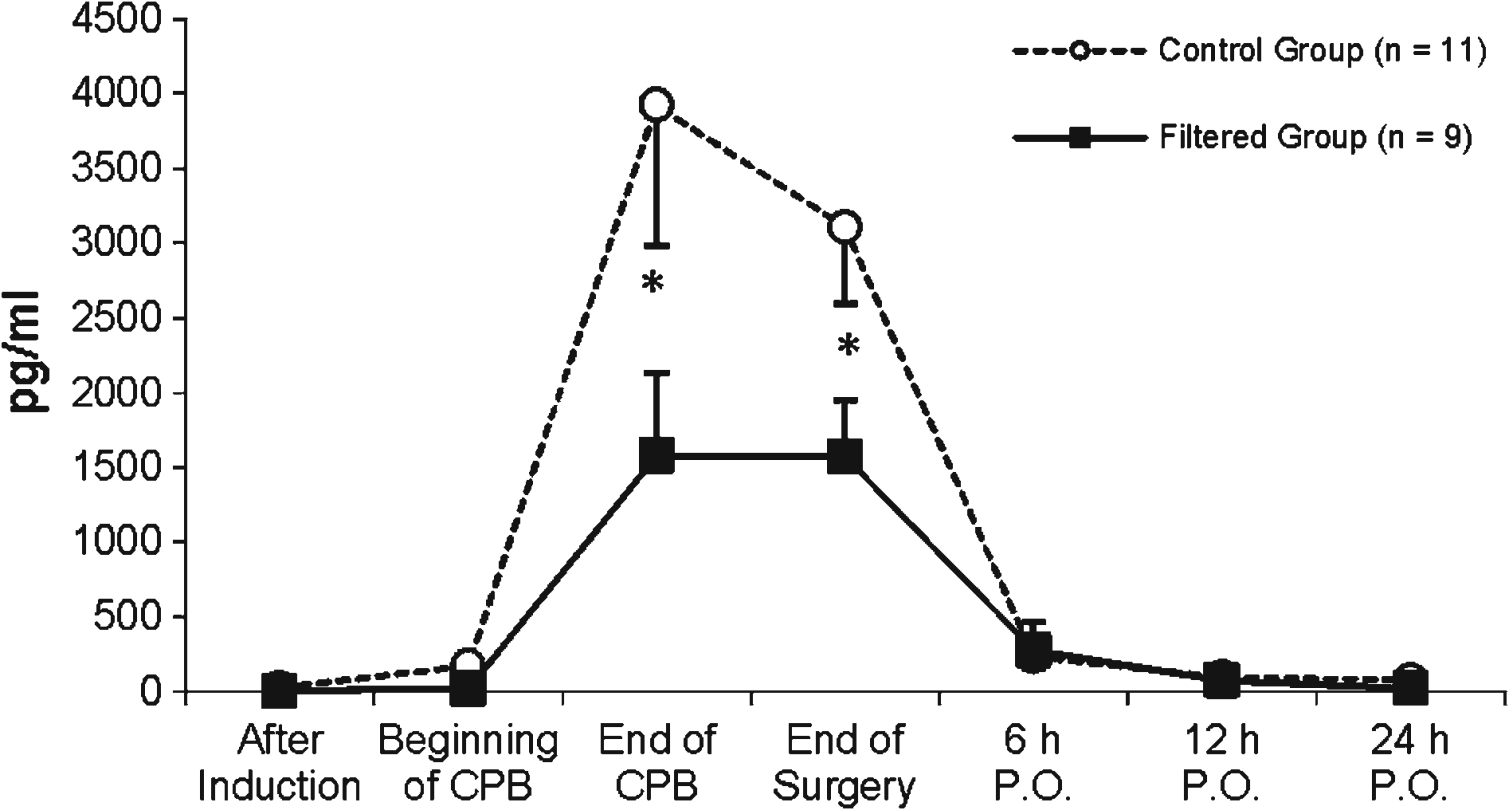
Changes in IL-10 in different groups (mean ± SEM). Comparison between groups by two-factor ANOVA for repeated measures: *p < 0.05 (statistically significant difference between groups).

As shown in Table 3, TNF-α and IL-1β serum levels did not present significant changes over time or between groups. For IL-6, IL-8, and IL-1rA, an increase over time was observed without differences between groups, the same for enzymes regulating cell degranulation, elastase, and MPO. On the other hand, IL-10 presented a significant difference between the control and filtered groups (*p* = 0.031). IL-10 serum levels were notably lower in the filtered group than in the control group from the end of CPB (*p* < 0.001) to the end of surgery (*p* = 0.002); thereafter, there was a reduction in these values with time in both groups (Fig. 6).

**Table 3:** Interleukins, Elastase, and MPO in Filtered and Control Groups (Mean ± SD)

	After induction	Beginning of CPB	End of CPB	End of surgery	6 h P.O.	12 h P.O.	24 h P.O.	*p* value
TNF-α (pg/mL)								0.846^a^
Control	5.77 ± 2.3	4.76 ± 1.4	11.12 ± 19.1	9.98 ± 13.1	5.77 ± 1.2	5.20 ± 1.4	4.74 ± 1.0	0.434^b^
Filtered	5.79 ± 1.2	4.49 ± 0.79	5.65 ± 2.80	6.17 ± 3.1	5.06 ± 3.5	6.38 ± 5.61	4.90 ± 2.9	
IL-1β (pg/mL)								0.271^a^
Control	7.41 ± 8.2	4.37 ± 3.6	4.99 ± 5.2	10.81 ± 23.7	14.00 ± 26.3	12.46 ± 20.6	14.66 ± 30.7	0.493^b^
Filtered	8.86 ± 11.3	6.92 ± 9.0	3.30 ± 0.2	5.21 ± 6.04	6.11 ± 6.16	5.32 ± 5.9	6.18 ± 7.15	
IL-6 (pg/mL)								0.511^a^
Control	62.01 ± 89.6	52.60 ± 84.6	197.41 ± 207.8	205.69 ± 167.2	180.4 ± 120.0	187.9 ± 112.0	110.4 ± 29.4	0.001^b^
Filtered	17.36 ± 34.8	33.90 ± 82.9	84.51 ± 78.3	122.59 ± 107.6	185.52 ± 147.9	203.5 ± 137.0	124.50 ± 120.2	
IL-8 (pg/mL)								0.757^a^
Control	15.77 ± 10.8	15.49 ± 12.2	42.07 ± 36.8	48.41 ± 48.6	38.33 ± 18.4	39.45 ± 14.03	29.90 ± 8.8	0.001^b^
Filtered	15.57 ± 14.5	21.31 ± 16.6	47.00 ± 36.54	49.04 ± 30.6	61.13 ± 52.1	48.39 ± 36.0	33.73 ± 25.0	
IL1-rA (pg/mL)								0.131^a^
Control	37.88 ± 50.9	49.15 ± 57.8	398.66 ± 534.5	818.9 ± 1,341.6	685.41 ± 863.4	114.40 ± 106.5	29.64 ± 221.1	0.001^b^
Filtered	11.91 ± 10.2	12.07 ± 13.0	38.70 ± 49.0	89.31 ± 77.3	490.13 ± 872	133.49 ± 240.4	11.63 ± 13.1	
Elastase (U/mL)								0.846^a^
Control	4.07 ± 2.56	3.65 ± 2.42	3.13 ± 1.84	3.33 ± 1.92	3.56 ± 2.39	3.46 ± 1.99	3.33 ± 1.89	0.001^b^
Filtered	4.91 ± 7.88	4.57 ± 7.01	3.68 ± 6.05	3.81 ± 6.47	4.92 ± 9.04	3.31 ± 4.8	3.86 ± 6.44	
MPO (ng/mL)								0.200^a^
Control	26.83 ± 15.9	225.31 ± 203.0	451.54 ± 384.7	418.46 ± 552.3	260.39 ± 228.3	154.29 ± 85.4	80.15 ± 133.7	0.001^b^
Filtered	29.71 ± 115.7	153.33 ± 200.8	808.74 ± 729.8	591.29 ± 662.3	452.62 ± 225.4	286.60 ± 174.5	118.90 ± 199.3	

*p* values = comparison between groups by two-factor ANOVA for repeated measures

^a^Difference between groups

^b^Variation over time by the Student–Newman–Keuls test

Regarding parameters derived from the chest helical CT on the first postoperative day, compared to preoperative values (Table 4), the filtered and control groups both presented increases in lung weight (*p* = 0.001 and *p* = 0.004, respectively). However, the results derived from entire lung volume (gas plus tissue) showed reductions over time in the control group (*p* = 0.002), but not in the filtered group (*p* = 0.083). These data reflect a decline in gas volume after surgery, but no differences between the groups were observed in either lung weight or lung volume (*p* = 0.379 and *p* = 0.220, respectively). In addition, the incremental changes in lung weight, represented as gains in lung tissue, were observed with no statistically significant difference between groups (*p* = 0,250), which can be attributed to increased extravascular lung water observed postoperatively.

**Table 4:** Global Analysis of the Entire Lungs by Computed Tomography Scan Assessment for the Control and Filtered Groups (Mean ± SD)

		Pre-op.	Post-op.	*p* value^a^ (over time)	*p* value^b^ (between groups)
Lung volume, gas + tissue (mL)	Control group	3,171 ± 896	2,499 ± 193	0.020	0.379
	Filtered group	2,834 ± 12,197	2,363 ± 170	0.083	
Volume of lung tissue (mL)	Control group	899 ± 201	1,046 ± 188	0.009	0.250
	Filtered group	734 ± 513	951 ± 666	0.002	
Gas volume (mL)	Control group	2,271 ± 859	1,453 ± 619	0.002	0.569
	Filtered group	2,100 ± 1,478	1,412 ± 992	0.008	
Lung weight (g)	Control group	900 ± 201	1,050 ± 189	0.004	0.220
	Filtered group	735 ± 166	938 ± 131	0.001	
Non-aereated lung volume (mL)	Control group	37.3 ± 27	252.6 ± 100	<0.001	0.376
	Filtered group	40.8 ± 39	282 ± 146	<0.001	
Poorly aerated lung volume (mL)	Control group	301.53 ± 111	486.51 ± 122	<0.001	0.184
	Filtered group	227.38 ± 98	378.87 ± 126	0.002	
Normally aerated lung (mL)	Control group	2,508 ± 558	1,609 ± 526	<0.001	0.599
	Filtered group	2,353 ± 923	1,518 ± 462	<0.001	

^a^Variation over time for the groups calculated by the Student–Newman–Keuls test

^b^Comparison between groups by two-factor ANOVA for repeated measures

## DISCUSSION

The results of this study showed that leukocyte filtration during CPB promoted a decrease in the neutrophil count, proportionally more evident than any change in the total leukocyte count, suggesting the effectiveness of the filter on activated polymorphonuclear cells. Reduced IL-10 expression in the filtered group was also observed. However, the beneficial effects on respiratory function were transient, with a reduced increase in the shunt fraction, suggesting a limited filtration capacity over the time it was used.

Regarding evaluation of the filter's effectiveness, the technique of its use during CPB can lead to different results in both clinical and experimental studies [7, 20–25]. Differences in filterability can be found when using one versus more than one filter, when the filter is used in the arterial versus the venous line or when it is used only part of the time versus for the total duration of CPB [8, 10, 26, 27]. With arterial line filtration, successful neutrophil reduction was time dependent, with limited improvement in lung function [8, 22]. On the other hand, a decrease in CPB flow can improve the efficiency of the filter in neutrophil depletion, resulting in diminished extravascular lung water and an absence of focal cellular injury signs as a result of reduced pulmonary sequestration of leukocytes, implying an improved arterial partial pressure of oxygen [26]. In clinical trials [28], flow variations imposed by the weight of individuals and surgical conditions, as well non-standard duration times for CPB, seemed to negatively affect an appropriate assessment of filter efficacy, compared with experimental models [26]. Such findings are probably responsible for the differences found between the current study and other reports [29]. We believe that definitive guidelines for when to use a leukocyte filter in the CPB circuit still remain to be determined, although some studies have discouraged its routine use [29, 30].

While the reduced PaO_2_/FiO_2_ ratio was unrelated to leukocyte filtration, the shunt fraction exhibited a less notable increase, not exceeding the early postoperative period, indicating better preservation of lung function. This observed effect on oxygenation could be related to a decrease in surfactant synthesis, resulting in lung collapse, especially in the dorsal part [31]. Depletion of activated cells can prevent endothelial injury, with reduced alveolar infiltration and a slight change in the vascular response mechanisms and pulmonary shunt [32]. However, the transience of the filtration effect is reinforced by the absence of more dramatic changes in the atelectasis grade found on the first postoperative day.

In that regard, post-CPB lung injury was characterized at CT scan as having a reduction in cephalocaudal dimensions, secondary to a decreased gas volume and increased tissue volume [33]. A postoperative volumetric loss pattern was observed in the control group but not in the filtered group. However, the drop of 22 % in total aeration in the control group was similar to the 17 % reduction in the filtered group. As the postoperative CT was performed on the first day after surgery, the result observed also reveals the transient effect of the leukocyte filtration. The clinical limitation of performing CT immediately after surgery, when the shunt fraction was greater in the control group, prevented the identification of differences in gas aeration patterns between the groups.

A similar evaluation of hemodynamic data was observed between groups, with increases in heart rate and cardiac output and decreases in systemic vascular resistance, without a significant change in PVR over the time. The artificial control of hemodynamic data by volume replacement and vasoactive drug infusion after CPB could prevent expression of the effect of leukocyte filtration in the cardiovascular system, as observed in other clinical studies of leukodepletion [27].

The reduced expression of IL-10 observed in the filtered group may represent a protective effect of this technique on the lungs, contributing to the transient effect of leukocyte filtration on the intrapulmonary shunt fraction. The anti-inflammatory activity of IL-10 is related to a decreased recognition of the surface antigen, with consequences for cellular adhesion, predisposing to an immunosuppressive state [12]. Levels of IL-10 are related to the severity of surgical trauma [13] and the sepsis state [34] and interfere with morbidity after coronary syndromes and myocarditis [35]. Corticosteroids can diminish pro-inflammatory cytokine release and increase blood IL-10 levels after CPB [36]. Nevertheless, in the current study, methylprednisolone was administered to both groups and so could not explain the observed difference between the groups. Thus, our results suggest that the filtered group was protected from the inflammatory insult of CPB, which could represent an advantage of this method, suggesting a smaller organic insult and reduced anti-inflammatory response. Furthermore, increased levels of IL-1rA can follow IL-10 elevation [12], as observed in the current study in the control group, suggesting a greater intensity of the anti-inflammatory response. On the other hand, the transient effect of filtration had no effect on the behavior of levels of the other interleukins. So, to provide more complete information on this topic, further investigations, with a sample size calculation based on the IL-10, could better discuss the relationship of this cytokine with leukodepletion, something not done by us.

Although we found higher elastase and myeloperoxidase levels after leukodepletion, this has previously been observed [7] and, as in our findings, did not result in a worsening of the oxygenation parameters. Conversely, decreased plasmatic elastase and pulmonary myeloperoxidase values were observed when filtration was done for a short period of time during CPB, thus resulting in a better oxygenation index (PaO_2_/FiO_2_) 24 h after surgery [9] and indicating that the filtration duration and the CPB apparatus were responsible for these results. In that sense, we believe that our duration time of filtration of 50 min during CPB may be responsible for the absence of more dramatic changes in values after CPB in the filtered group, which could lead to statistical significance.

Besides filter characteristics, additional limitations of this study are related to sample size, which was calculated for evaluation of neutrophil count. Although neutrophil counts were decreased, the time dependence of the filter's efficiency, and variations in patients and CPB conditions, resulted in only a transient impact on pulmonary function and release of inflammatory mediators. Additional studies of filtered and non-filtered cells, and improvements in the technological characteristics of the leukocyte filter, could result in innovation and better clinical results. Considering what was our primary objective, the benefit observed could represent one possible strategy to be used along with other methods, such as decreasing intraoperative fluid overload and reducing blood transfusion and operative length, in order to decrease overall morbidity associated to surgical procedures using CPB.

In conclusion, leukocyte filtration during cardiopulmonary bypass, when using an arterial line filter with a flow of up to 5 L/min m^2^ during the entire duration of CPB, results in an effective neutrophil sequestration up to 50 min of the machine operation and in decreased IL-10 serum levels up to the end of surgery, protecting the lungs only temporarily against acute injury related to CPB.
